# Safety and Effectiveness of Behavioural Activation for Chronic Pain: A Protocol for a Systematic Review and Meta-Analysis of Randomised Controlled Trials

**DOI:** 10.3390/nursrep16080286

**Published:** 2026-08-17

**Authors:** Fangyuan Zheng, Laura Hynes, Richard J. Gray, Irene Ngune, Jacqueline Sin, Martin Jones

**Affiliations:** 1 School of Nursing and Midwifery, Edith Cowan University, South West Campus, 585 Robertson Drive, Bunbury, WA 6230, Australia; fzheng0@our.ecu.edu.au (F.Z.); l.hynes@ecu.edu.au (L.H.); i.ngune@ecu.edu.au (I.N.); 2 Deputy Vice-Chancellor (Research & Industry Engagement) Office, La Trobe University, Melbourne, VIC 3086, Australia; r.gray@latrobe.edu.au; 3 School of Nursing, The Hong Kong Polytechnic University, Hong Kong SAR, China; jacqueline-ph.sin@polyu.edu.hk

**Keywords:** behavioural activation, chronic pain, pain intensity, adverse events, systematic review, meta-analysis, protocol, randomised controlled trial, nursing

## Abstract

**Background/Objectives**: Chronic pain is a prevalent and complex condition associated with substantial physical, psychological, and social burden. Behavioural activation (BA), a structured psychological intervention originally developed for depression, may be relevant to chronic pain through its focus on reducing behavioural avoidance and increasing engagement in meaningful activities. Although BA has been applied in studies involving people with chronic pain, the safety and effectiveness of BA in this population have not been comprehensively synthesised. This systematic review and meta-analysis will examine the safety and effectiveness of BA for people with chronic pain. **Methods**: A systematic review and meta-analysis of literature evidence will be conducted. This protocol was developed in accordance with the Preferred Reporting Items for Systematic Review and Meta-Analysis Protocols (PRISMA-P) and prospectively registered with PROSPERO (CRD420261379007). Randomised controlled trials (RCTs) evaluating BA or BA-based interventions for individuals with chronic pain will be eligible. MEDLINE, Cumulative Index to Nursing and Allied Health Literature (CINAHL), PsycINFO, Emcare, Web of Science Core Collection, Scopus and the Cochrane Central Register of Controlled Trials (CENTRAL), trial registries, and citation tracking will be searched. Two reviewers will independently screen records, extract data, and assess risk of bias using the Cochrane Risk of Bias 2 (RoB 2) tool. Where appropriate, meta-analysis will be conducted using a random-effects model. Pain intensity will be the primary outcome. Reported adverse events and other safety outcomes will be extracted and synthesised. Certainty of evidence will be assessed using the Grading of Recommendations Assessment, Development, and Evaluation (GRADE) approach. **Results**: Not applicable; this is a protocol. Findings will be synthesised as specified in the Methods. **Conclusions**: The review will evaluate the effectiveness of BA with respect to pain intensity among people with chronic pain and examine what is known about its safety based on reported adverse events and other safety outcomes in existing trials. The review is expected to clarify the extent, consistency, and certainty of the randomised evidence on the effectiveness and safety of behavioural activation interventions for chronic pain and to identify gaps in adverse-event reporting and safety monitoring. Findings may inform clinical decision-making, intervention adaptation, and future research on BA for chronic pain.

## 1. Introduction

Chronic pain is commonly defined as pain that persists or recurs for more than three months [1]. It is associated with substantial physical, psychological, and social burdens, including functional disability, reduced quality of life, and emotional distress [2,3,4]. In addition to biological mechanisms, psychological and behavioural factors contribute to the maintenance of chronic pain. Pain-related avoidance behaviours and reduced engagement in meaningful activities may contribute to a cycle of disability and distress, as described in the fear-avoidance model of pain [5,6].

Behavioural activation (BA) is a structured intervention first developed for depression. It aims to reduce avoidance behaviours and support greater engagement in meaningful or valued activities [7]. Given its emphasis on behavioural processes, BA may be relevant to chronic pain. It may influence pain-related outcomes indirectly, through improvements in mood, activity engagement, and functioning, and directly, by interrupting cycles of avoidance, deconditioning, and disability [8]. BA may also be relatively simple and scalable compared with some more complex psychological interventions, supporting potential implementation across diverse clinical settings. Evidence from depression research provides support for the potential scalability of BA. The Cost and Outcome of Behavioural Activation versus Cognitive Behavioural Therapy for Depression (COBRA) trial evaluated BA against cognitive behavioural therapy in 440 adults with moderate-to-severe depression, using a randomised controlled non-inferiority design [9]. BA was delivered by junior mental health workers after brief training, whereas cognitive behavioural therapy was delivered by qualified psychological therapists [9]. BA was reported to be similarly effective in reducing depressive symptoms and more cost-effective than cognitive behavioural therapy, although its effectiveness and safety in chronic pain require direct evaluation [9].

Existing evidence on BA for chronic pain remains fragmented. Studies differ in populations, pain conditions, intervention formats, comparator types, outcome measures, and follow-up periods. Previous research has also included heterogeneous designs, including randomised controlled trials (RCTs), uncontrolled studies, and case studies, which limits the extent to which intervention effects can be estimated from the existing literature. In addition, BA may overlap conceptually and practically with activity scheduling, activity monitoring, pleasant activities, and other behavioural components used in psychological or multidisciplinary pain interventions. This creates methodological challenges for identifying eligible studies and distinguishing BA-focused interventions from broader psychosocial intervention packages.

A previous systematic scoping review mapped the use of BA to manage pain and identified 15 included papers from 551 screened records, comprising three RCTs, nine uncontrolled trials, and three case studies [10]. The review showed that BA had been applied across a range of pain-related conditions and delivery formats, but only two studies reported pain as the primary outcome [10]. As a scoping review, its purpose was to describe how BA had been used in relation to pain, rather than to estimate pooled intervention effects. Accordingly, no meta-analysis was conducted, and the certainty of evidence was not assessed using the Grading of Recommendations Assessment, Development, and Evaluation (GRADE) approach. The review also found that the three included RCTs were at high risk of bias and that none of the included studies reported adverse events or identified potential risks [10]. The overall magnitude and certainty of effects, and the extent to which adverse events have been assessed or reported, remain unclear. Preliminary scoping searches suggest that additional RCTs have been completed since that review. Therefore, a systematic review and meta-analysis using prespecified eligibility criteria, Cochrane Risk of Bias 2 (RoB 2) assessment, and GRADE certainty assessment is needed to clarify the effectiveness and safety of BA for chronic pain.

Prospective registration and peer-reviewed protocol publication serve complementary functions in systematic review methodology. PROSPERO registration establishes a time-stamped public record of the planned review, whereas protocol publication provides a more detailed account of the methodological decisions underpinning the review, including eligibility decision rules, intervention definitions, outcome-selection procedures, complete search strategies, risk-of-bias assessment, GRADE certainty assessment, and safety synthesis. This is particularly relevant for the present review because the existing evidence base includes heterogeneous study designs, variable pain-related outcomes, limited randomised trial evidence, and unclear adverse-event reporting. In addition, behavioural activation overlaps with activity-based behavioural components and may be delivered either as a standalone intervention or within integrated care packages. A transparent protocol will support reproducibility and provide a reference point against which the conduct and reporting of the completed review can be assessed, thereby reducing the risk of selective outcome reporting and post hoc methodological decisions [11,12].

This systematic review and meta-analysis aim to evaluate the effectiveness of BA, compared with control or alternative interventions, in reducing pain intensity in individuals with chronic pain, and to assess safety by synthesising the adverse events and other safety outcomes that have been reported.

## 2. Materials and Methods

The methods of this review are informed by the Cochrane Handbook for Systematic Reviews of Interventions [13]. The completed review will be reported in accordance with the Preferred Reporting Items for Systematic Reviews and Meta-Analyses (PRISMA) 2020 statement [14].

### 2.1. Protocol Design and Registration

This protocol was developed and reported in accordance with the Preferred Reporting Items for Systematic Review and Meta-Analysis Protocols (PRISMA-P) 2015 statement [11,12] (File S3). The review was registered with the International Prospective Register of Systematic Reviews (PROSPERO; CRD420261379007). Any important protocol amendments will be documented in PROSPERO and reported in the completed review.

### 2.2. Review Question

The review question is the following: among individuals with chronic pain, what is the effectiveness of BA, compared with control or alternative interventions, in reducing pain intensity, and what is known about its safety based on reported adverse events and other safety outcomes?

The PICO framework was used to structure the review question. Population: individuals with chronic pain. Intervention: behavioural activation interventions, including interventions in which activation-based strategies are a core therapeutic component. Comparator: usual care, waitlist, no treatment, attention control, education, or active psychological, behavioural, or pharmacological comparators. Outcomes: pain intensity is the primary outcome, while the safety outcomes comprise adverse events, serious adverse events, withdrawals due to adverse events, and safety monitoring procedures.

### 2.3. Eligibility Criteria

Eligibility criteria will be defined using the population, intervention, comparator, outcome, and study design framework (PICOS). The criteria are summarised in Table 1.

In this review, behavioural activation (BA) is defined as a structured psychological intervention that aims to increase engagement in meaningful and rewarding activities while reducing avoidance and activity withdrawal [7]. Eligible interventions include those explicitly described as BA or behavioural activation treatment, as well as interventions in which activity scheduling, graded engagement in valued activities, reduction of avoidance, or re-engagement with meaningful activities form a core therapeutic component.

For multicomponent interventions, BA will be considered a primary intervention component when the trial report, intervention manual, or protocol indicates that activation-based strategies are central to the treatment rationale, session content, or therapeutic procedures. Interventions will not be eligible solely because they include general advice to remain active, exercise prescription, pacing advice, or pain education, unless these elements are delivered within a structured activation-based approach. Where the eligibility of an intervention is unclear, it will be resolved using the two-reviewer process described in Section 2.5.

Published and unpublished studies, including trial registry records where sufficient methodological and outcome information is available, will be considered for inclusion. Where registry records or unpublished reports do not contain sufficient outcome data, study authors may be contacted for clarification. No language restrictions will be applied. Non-English records will be screened using translation support where required. Where potentially eligible, non-English full-text reports are identified and translation tools—where necessary with assistance from speakers of the relevant language—will be used to support eligibility assessment and data extraction.

### 2.4. Information Sources and Search Strategy

A comprehensive search will be conducted from database inception to the final search date in MEDLINE, the Cumulative Index to Nursing and Allied Health Literature (CINAHL) and PsycINFO (all via EBSCOhost), Emcare (via Ovid), Web of Science Core Collection (Clarivate), and Scopus (Elsevier). The Cochrane Central Register of Controlled Trials (CENTRAL) will also be searched as a dedicated trials database. Supplementary searches will include trial registries, backward citation tracking of eligible studies and relevant reviews, and forward citation tracking using Google Scholar. Trial registries will include ClinicalTrials.gov, the World Health Organization International Clinical Trials Registry Platform (WHO ICTRP), the Australian New Zealand Clinical Trials Registry (ANZCTR), and the International Standard Randomised Controlled Trial Number (ISRCTN) registry.

The search strategy was developed using a combination of database-specific controlled vocabulary, including Medical Subject Headings (MeSH) where available, and free-text terms related to chronic pain and behavioural activation. Terms for behavioural activation will include British and American spellings and related behavioural activation components, including activity scheduling, activity monitoring, pleasant activities, activity engagement, and meaningful activities. Outcome terms and study design filters will not be applied in the database search to maximise sensitivity; eligibility restrictions to RCTs will be applied during screening. The search strategy was developed in consultation with an experienced research librarian and informed by the Peer Review of Electronic Search Strategies (PRESS) guideline [15]. The complete search strategy for each electronic database, together with the search terms used for each trial registry, is provided in Supplementary File S1.

### 2.5. Study Selection

All identified records will first be managed in Zotero (version 9.0.6; Corporation for Digital Scholarship, Falls Church, VA, USA) to remove duplicate records, before being transferred to Covidence systematic review software (Veritas Health Innovation, Melbourne, Australia) for additional duplicate checking and screening. Screening will be undertaken in two sequential steps. In the first step, two reviewers will assess titles and abstracts independently against the prespecified eligibility criteria. In the second step, full-text reports judged potentially relevant will be assessed independently by two reviewers. Any disagreements will be discussed between reviewers, with a third reviewer consulted if consensus cannot be reached. Reasons for exclusion at full-text screening will be recorded and presented in the completed review using a PRISMA 2020 flow diagram.

Multiple reports of the same underlying trial will be identified and collated. Trial registration numbers, author lists, settings, sample sizes, intervention details, and outcome time points will be compared to identify related reports. Where uncertainty remains, study authors may be contacted for clarification.

### 2.6. Data Extraction

Two reviewers will independently extract data using a prespecified extraction form, which will be piloted on a subset of included studies and revised if needed. Extracted data will include study characteristics, participant characteristics, chronic pain condition, intervention components, comparator details, outcome measures, time points, outcome scales, direction of scoring, numbers of participants analysed, means, standard deviations, mean changes, standard errors, confidence intervals, effect estimates where reported, and funding or conflict-of-interest information.

Pain intensity will be the primary outcome and will be extracted separately from pain interference. Where a study reports more than one pain intensity measure at the same point, one measure will be selected using a prespecified hierarchy, independent of the observed results, consistent with Cochrane Handbook guidance on selecting among multiple measures of the same outcome domain [13]. The hierarchy will prioritise validated unidimensional pain-intensity measures, with Numeric Rating Scale scores selected first, followed by Visual Analogue Scale scores, Brief Pain Inventory pain severity scores, and other validated pain intensity measures. Pain interference outcomes, including the Brief Pain Inventory Interference Scale, will be recorded separately and will not be treated as pain intensity outcomes.

Intervention data will include BA components, delivery mode, provider, number and length of sessions, intervention duration, frequency, and self-directed practice. Safety data will include adverse events, serious adverse events, withdrawals due to adverse events, and information on whether safety outcomes were assessed, monitored, reported, explicitly reported as absent, unclear, or not reported. Where required, standard errors, confidence intervals, or other reported statistics will be converted to standard deviations or other metrics needed for meta-analysis using established methods. The planned data extraction form is provided as File S2 in the Supplementary Materials. Discrepancies in extracted data will be resolved through discussion between the two reviewers; if consensus cannot be reached, a third reviewer will be consulted. Missing or unclear data may be clarified by contacting the study authors.

### 2.7. Risk of Bias Assessment

Two reviewers will independently assess risk of bias using the Cochrane Risk of Bias 2 (RoB 2) tool for randomised trials [16]. The assessment will consider bias arising from the randomisation process, bias due to deviations from intended interventions, bias due to missing outcome data, bias in measurement of the outcome, and bias in selection of the reported result. Each domain, and the overall judgement, will be rated as low risk of bias, some concerns, or high risk of bias. Disagreements will be resolved through reviewer discussion, with a third reviewer consulted where required.

### 2.8. Data Synthesis and Meta-Analysis

A meta-analysis will be conducted if included studies are sufficiently similar in terms of population, intervention, comparator, outcome measurement, and assessment time point. For continuous pain intensity outcomes, standardised mean differences (SMDs) with 95% confidence intervals (CIs) will be calculated when studies use different scales. Mean differences (MDs) will be calculated where outcomes are measured using the same scale. A random-effects model will be used as the primary meta-analytic approach because clinical and methodological heterogeneity is anticipated across pain conditions, intervention formats, comparators, and outcome measures. Fixed-effect models will be considered only in sensitivity analyses where studies are judged to be sufficiently comparable in terms of population, intervention, comparator, outcome measurement, and assessment timing. Where appropriate and statistically feasible, prediction intervals will be reported to describe the range within which the effect of a future comparable study would be expected to lie [13]. Statistical heterogeneity will be assessed using Cochran’s Q test and the I^2^ statistic. Where necessary, means and standard deviations will be estimated from medians and other summary statistics using established methods [17,18].

For the primary analysis, outcome data will be extracted at post-treatment, defined as the assessment conducted closest to completion of the behavioural activation intervention. If more than one post-treatment assessment is reported, the one closest to the end of treatment will be selected.

Follow-up assessments will be considered in secondary analyses and categorised as short term (≤3 months after treatment), medium term (>3 to 6 months after treatment), and long term (>6 months after treatment). All eligible follow-up time points will be extracted. Where multiple assessments are reported within the same follow-up category, the assessment closest to the end of the relevant period will be selected for quantitative synthesis; for long-term follow-up, the longest available assessment will be selected.

Where sufficient data are available, subgroup analyses may be conducted to explore potential sources of heterogeneity, including intervention format, pain type, population characteristics such as age group or sex, and whether BA is delivered as a standalone intervention or within an integrated package of care. Sensitivity analyses may be conducted by excluding studies at high risk of bias or studies requiring data estimation. Publication bias will be assessed using funnel plots and Egger’s regression test if at least 10 studies are included in a meta-analysis [19]. If meta-analysis is not feasible, findings will be synthesised narratively, with reporting informed by the Synthesis Without Meta-analysis (SWiM) guideline where applicable [20]. Analyses will be conducted using Review Manager (RevMan, version 11.5.0; Cochrane, London, UK) where appropriate.

### 2.9. Safety Synthesis

For this review, adverse events will be defined as any unfavourable or unintended sign, symptom, or experience reported during the intervention or follow-up period, whether or not it is judged by study authors to be related to the intervention. Serious adverse events will include events reported by study authors as serious, including death, life-threatening events, hospitalisation, persistent or significant disability, or other medically important events. Withdrawals due to adverse events will refer to participants who discontinue the intervention or study because of an adverse event. Safety monitoring procedures will refer to any procedures used by study authors to identify, monitor, record, or report adverse events.

Safety outcomes will be synthesised separately from effectiveness outcomes. Reported adverse events, serious adverse events, and withdrawals due to adverse events will be treated as safety outcomes. Safety monitoring procedures will be extracted separately as process information describing how harms were assessed, monitored, and reported. Where event counts by treatment group are extractable and outcome definitions are sufficiently comparable, dichotomous safety outcomes, such as adverse events, serious adverse events, and withdrawals due to adverse events, will be synthesised quantitatively using risk ratios with 95% CIs; otherwise, they will be summarised descriptively.

For each study, we will extract whether adverse-event monitoring or reporting was prespecified in the trial protocol or registration, how adverse events and serious adverse events were defined, how events were ascertained, whether events were reported separately for each arm, and whether relatedness to the intervention was assessed. Within the prespecified safety-outcome domain, we will specifically extract serious adverse events, including deaths or hospitalisations where reported, withdrawals or discontinuations due to adverse events, and adverse events of particular relevance to this population and intervention, including worsening of pain, psychological distress or worsening mental state, and serious psychological deterioration, including suicidal ideation or self-harm where reported. For each included study, safety information will be checked across the main trial report, Supplementary Materials, trial protocol, and trial registration, where available. Planned safety monitoring or adverse-event reporting in the protocol or registration will be compared with what is reported in the final trial publication.

Explicit reports of no adverse events will be recorded separately from missing or unclear safety reporting. Poor or absent reporting of safety outcomes will be treated as an important finding of the review.

### 2.10. Certainty of Evidence

The certainty of evidence for the primary outcome and key safety outcomes will be assessed using the GRADE approach [21]. For RCTs, certainty will initially be considered high and may be downgraded for risk of bias, inconsistency, indirectness, imprecision, and publication bias. Certainty will be rated as high, moderate, low, or very low. GRADEpro Guideline Development Tool (GRADEpro GDT; McMaster University and Evidence Prime Inc., Hamilton, ON, Canada) will be used to prepare Summary of Findings tables for the main outcomes. Two reviewers will conduct GRADE assessments independently, with disagreements resolved through discussion or consultation with a third reviewer.

## 3. Results

Not applicable; this manuscript reports a protocol. The completed systematic review will present the study selection process using a PRISMA 2020 flow diagram, summarise study and intervention characteristics, report risk of bias assessments, synthesise pain intensity and safety outcomes, and present certainty of evidence using Summary of Findings tables where appropriate.

## 4. Conclusions

This protocol describes the planned methods for a systematic review and meta-analysis evaluating the safety and effectiveness of BA for chronic pain. The completed review will clarify the effects of BA on pain intensity among people with chronic pain and examine what is known about its safety based on reported adverse events and other safety outcomes in existing RCTs.

The planned review will provide a prespecified and methodologically transparent synthesis of randomised evidence on behavioural activation interventions for chronic pain. By integrating effectiveness outcomes with adverse-event reporting and safety monitoring procedures, the review is expected to clarify the current evidence base and identify methodological and reporting gaps relevant to future research and clinical implementation.

## Figures and Tables

**Table 1:** Eligibility criteria for the planned systematic review.

Domain	Inclusion Criteria	Exclusion Criteria
Population	Individuals of any age with chronic pain, as defined by the authors of the primary study, with or without comorbid physical or mental health conditions.	Studies in which chronic pain is not a clear population characteristic or intervention focus.
Intervention	BA or BA-based interventions delivered in any format, setting, or provider arrangement. Multi-component interventions will be eligible if BA is the primary component, as described by the study authors.	Interventions in which BA is not clearly identified, is a minor component, or cannot be meaningfully distinguished from other psychosocial components. Studies will also be excluded if BA is delivered primarily to address another condition and chronic pain is not a clear focus of the intervention.
Comparator	Any comparator, including usual care, waitlist, no treatment, attention control, psychological intervention, pharmacological care, or another active comparator.	None based on comparator type.
Outcomes	Primary outcome: pain intensity measured using a prespecified acceptable pain intensity measure, including VAS, NRS, BPI pain intensity components, or another measure considered acceptable for this review. Safety outcomes include adverse events, serious adverse events, withdrawals due to adverse events, and statements on safety assessment or monitoring.	Studies that do not report pain intensity or do not provide data that can be extracted or requested.
Study design	Randomised controlled trials.	Non-randomised studies, observational studies, qualitative studies, reviews, editorials, commentaries, and case reports.
Other limits	No restrictions will be applied by age, publication date, publication status, language, geography, or setting.	None based on these criteria.

Note: BA, Behavioural Activation; BPI, Brief Pain Inventory; NRS, Numeric Rating Scale; VAS, Visual Analogue Scale.

## Data Availability

No datasets were generated or analysed for this protocol. Data availability for the completed review will be described in the final review manuscript.

## Supplementary Materials

The following supporting information can be downloaded at: https://www.mdpi.com/article/10.3390/nursrep16080286/s1, File S1: Complete Search Strategy; File S2: Planned Data Extraction Form; File S3: PRISMA-P 2015 checklist.

## Public Involvement Statement

No public involvement in any aspect of this research.

## Guidelines and Standards Statement

This manuscript was drafted in accordance with the Preferred Reporting Items for Systematic Review and Meta-Analysis Protocols (PRISMA-P) 2015 statement. The completed review will be reported in accordance with PRISMA 2020.

## Abbreviations

The following abbreviations are used in this manuscript:

ANZCTR	Australian New Zealand Clinical Trials Registry
BA	Behavioural Activation
BPI	Brief Pain Inventory
CENTRAL	Cochrane Central Register of Controlled Trials
CI	Confidence Interval
CINAHL	Cumulative Index to Nursing and Allied Health Literature
COBRA	Cost and Outcome of Behavioural Activation versus Cognitive Behavioural Therapy for Depression
GRADE	Grading of Recommendations Assessment, Development, and Evaluation
GRADEpro GDT	GRADEpro Guideline Development Tool
ISRCTN	International Standard Randomised Controlled Trial Number
MeSH	Medical Subject Headings
MD	Mean Difference
NRS	Numeric Rating Scale
PICOS	Population, Intervention, Comparator, Outcome, and Study design
PRESS	Peer Review of Electronic Search Strategies
PRISMA	Preferred Reporting Items for Systematic Reviews and Meta-Analyses
PRISMA-P	Preferred Reporting Items for Systematic Review and Meta-Analysis Protocols
PROSPERO	International Prospective Register of Systematic Reviews
RCT	Randomised Controlled Trial
RoB 2	Cochrane Risk of Bias 2 tool
SMD	Standardised Mean Difference
SWiM	Synthesis Without Meta-analysis
VAS	Visual Analogue Scale
WHO ICTRP	World Health Organization International Clinical Trials Registry Platform
