# Supplementary material for: Safety and Effectiveness of Behavioural Activation for Chronic Pain: A Protocol for a Systematic Review and Meta-Analysis of Randomised Controlled Trials
Source: Nurs Rep. 2026 Aug 17;16(8):286. doi: 10.3390/nursrep16080286 (PMC13516624; doi:10.3390/nursrep16080286)
Supplement: Supplementary file 1 [file nursrep-16-00286-s001.zip › File S1-Complete Search Strategy.pdf]

Supplementary File S1:

MEDLINE Search Strategy

Database/platform: MEDLINE via EBSCOhost
Date searched: 4 May 2026
Limits applied: None
Search result: 873 records

Search line	Search terms	Results
**S1**	MH "Chronic Pain"	29,465
**S2**	TX chronic pain	142,884
**S3**	TX persistent pain	24,341
**S4**	TX long-term pain	12,738
**S5**	TX chronic primary pain	3,180
**S6**	TX chronic secondary pain	1,285
**S7**	TX fibromyalgia	21,968
**S8**	TX musculoskeletal pain	24,111
**S9**	TX neuropathic pain	46,482
**S10**	TX cancer pain	32,032
**S11**	TX back pain	105,510
**S12**	TX chronic N3 pain	137,441
**S13**	TX behavioural activation	1,404
**S14**	TX behavioral activation	6,159
**S15**	TX activity scheduling	442
**S16**	TX pleasant activities	354
**S17**	TX activity engagement	10,372
**S18**	TX activity monitoring	22,969
**S19**	TX behavior*	2,535,090
**S20**	TX behaviour*	595,708
**S21**	S1 OR S2 OR S3 OR S4 OR S5 OR S6 OR S7 OR S8 OR S9 OR S10 OR S11 OR S12	304,139
**S22**	S15 OR S16 OR S17 OR S18	33,921
**S23**	S19 OR S20	2,902,029
**S24**	S22 AND S23	9,570
**S25**	S13 OR S14 OR S24	16,752
**S26**	S21 AND S25	873

CINAHL Search Strategy

Database/platform: CINAHL Ultimate via EBSCOhost
Date searched: 4 May 2026
Limits applied: None
Search result: 5352 records

Search line	Search terms	Results
**S1**	MH "Chronic Pain"	29,929
**S2**	TX chronic pain	127,115
**S3**	TX persistent pain	21,891
**S4**	TX long-term pain	14,466
**S5**	TX chronic primary pain	3,629
**S6**	TX chronic secondary pain	1,344
**S7**	TX fibromyalgia	22,816
**S8**	TX musculoskeletal pain	30,842
**S9**	TX neuropathic pain	21,990
**S10**	TX cancer pain	39,968
**S11**	TX back pain	107,537
**S12**	TX chronic N3 pain	122,654
**S13**	TX behavioural activation	6,074
**S14**	TX behavioral activation	6,074
**S15**	TX activity scheduling	2,204
**S16**	TX pleasant activities	1,583
**S17**	TX activity engagement	28,584
**S18**	TX activity monitoring	19,316
**S19**	TX behavior*	1,132,533
**S20**	TX behaviour*	439,226
**S21**	S1 OR S2 OR S3 OR S4 OR S5 OR S6 OR S7 OR S8 OR S9 OR S10 OR S11 OR S12	261,314
**S22**	S15 OR S16 OR S17 OR S18	49,587
**S23**	S19 OR S20	1,358,533
**S24**	S22 AND S23	35,182
**S25**	S13 OR S14 OR S24	40,348
**S26**	S21 AND S25	5,352

APA PsycInfo Search Strategy

Database/platform: APA PsycInfo via EBSCOhost
Date searched: 4 May 2026
Limits applied: None
Search result: 203 records

Search line	Search terms	Results
**S1**	DE "Chronic Pain"	19,752
**S2**	TX chronic pain	31,594
**S3**	TX persistent pain	2,626
**S4**	TX long-term pain	1,635
**S5**	TX chronic primary pain	565
**S6**	TX chronic secondary pain	195
**S7**	TX fibromyalgia	4,386
**S8**	TX musculoskeletal pain	3,502
**S9**	TX neuropathic pain	7,469
**S10**	TX cancer pain	4,457
**S11**	TX back pain	8,788
**S12**	TX chronic N3 pain	30,978
**S13**	TX behavioural activation	5,761
**S14**	TX behavioral activation	5,761
**S15**	TX activity scheduling	323
**S16**	TX pleasant activities	394
**S17**	TX activity engagement	7,606
**S18**	TX activity monitoring	2,801
**S19**	TX behavior*	1,964,796
**S20**	TX behaviour*	288,514
**S21**	S1 OR S2 OR S3 OR S4 OR S5 OR S6 OR S7 OR S8 OR S9 OR S10 OR S11 OR S12	48,345
**S22**	S15 OR S16 OR S17 OR S18	10,970
**S23**	S19 OR S20	2,085,103
**S24**	S22 AND S23	5,169
**S25**	S13 OR S14 OR S24	10,784
**S26**	S21 AND S25	203

Emcare Search Strategy

Database/platform: Emcare via Ovid
Date searched: 4 May 2026
Limits applied: None
Search result: 118 records

Search line	Search terms	Results
**1**	chronic pain/	29,532
**2**	(chronic pain or persistent pain or long-term pain or chronic primary pain or chronic secondary pain or fibromyalgia or musculoskeletal pain or neuropathic pain or cancer pain or back pain).mp.	213,903
**3**	(chronic adj3 pain).mp.	107,459
**4**	1 or 2 or 3	231,799
**5**	(behavioural activation or behavioral activation).mp.	2,978
**6**	((activity scheduling or pleasant activities or activity engagement or activity monitoring) and (behavior* or behaviour*)).mp.	1,186
**7**	5 or 6	4,102
**8**	4 and 7	118

Web of Science Core Collection Search Strategy

Database/platform: Web of Science Core Collection
Date searched: 3 July 2026
Limits applied: None
Search result: 193 records

Search line	Search terms	Results
**S1**	TS=("chronic pain")	78766
**S2**	TS=("persistent pain")	9361
**S3**	TS=("long-term pain")	1982
**S4**	TS=("chronic primary pain")	175
**S5**	TS=("chronic secondary pain")	30
**S6**	TS=(fibromyalgia)	25486
**S7**	TS=("musculoskeletal pain")	17,600
**S8**	TS=("neuropathic pain")	50,643
**S9**	TS=("cancer pain")	16,146
**S10**	TS=("back pain")	104,209
**S11**	TS=(chronic NEAR/3 pain)	128,991
**S12**	TS=("behavioural activation")	838
**S13**	TS=("behavioral activation")	4,379
**S14**	TS=("activity scheduling")	555
**S15**	TS=("pleasant activities")	173
**S16**	TS=("activity engagement")	1806
**S17**	TS=("activity monitoring")	5361
**S18**	TS=(behavior*)	5,233,521
**S19**	TS=(behaviour*)	1,231,001
**S20**	S1 OR S2 OR S3 OR S4 OR S5 OR S6 OR S7 OR S8 OR S9 OR S10 OR S11	279203
**S21**	S14 OR S15 OR S16 OR S17	7880
**S22**	S18 OR S19	6249277
**S23**	S21 AND S22	2032
**S24**	S12 OR S13 OR S23	7042
**S25**	S20 AND S24	193

Scopus Search Strategy

Database/platform: Scopus
Date searched: 3 July 2026
Limits applied: None
Search result: 191 records

Search line	Search terms	Results
**#1**	TITLE-ABS-KEY("chronic pain" OR "persistent pain" OR "long-term pain" OR "chronic primary pain" OR "chronic secondary pain" OR fibromyalgia OR "musculoskeletal pain" OR "neuropathic pain" OR "cancer pain" OR "back pain" OR (chronic W/3 pain))	355612
**#2**	TITLE-ABS-KEY("behavioural activation" OR "behavioral activation")	4226
**#3**	TITLE-ABS-KEY("activity scheduling" OR "pleasant activities" OR "activity engagement" OR "activity monitoring")	10407
**#4**	TITLE-ABS-KEY (behavior* OR behaviour*)	7497060
**#5**	#3 AND #4	2548
**#6**	#2 OR #5	6696
**#7**	#1 AND #6	191

Cochrane Library Search Strategy

Database/platform: Cochrane Library, including CENTRAL trial records
Date searched: 4 May 2026
Limits applied: None
Search result: 97 records overall; 76 trial records

Search line	Search terms	Results
**#1**	MeSH descriptor: [Chronic Pain] explode all trees	5,273
**#2**	"chronic pain" OR "persistent pain" OR "long-term pain" OR "chronic primary pain" OR "chronic secondary pain" OR fibromyalgia OR "musculoskeletal pain" OR "neuropathic pain" OR "cancer pain" OR "back pain"	48,739
**#3**	#1 OR #2	48,739
**#4**	"behavioural activation" OR "behavioral activation"	1,830
**#5**	"activity scheduling" OR "pleasant activities" OR "activity engagement" OR "activity monitoring"	1,035
**#6**	behavioral OR behavioural	95,421
**#7**	#4 OR (#5 AND #6)	2,098
**#8**	#3 AND #7	97
Note: Only CENTRAL trial records were exported for screening. Cochrane Reviews and Cochrane Protocols displayed in the interface were not exported as screening records but may be used for background information or citation checking where relevant.		

ClinicalTrials.gov Search Strategy

Database/platform: ClinicalTrials.gov
Date searched: 4 May 2026
Limits applied: None
Search result: 50 records before deduplication across searches

Search 1	Results
**Condition or disease:**	32
"chronic pain" OR "persistent pain" OR "long-term pain" OR "chronic primary pain" OR "chronic secondary pain" OR fibromyalgia OR "musculoskeletal pain" OR "neuropathic pain" OR "cancer pain" OR "back pain"	
**Intervention/treatment:**	
"behavioral activation" OR "behavioural activation"	
**Search 2**	
**Condition or disease:**	18
"chronic pain" OR "persistent pain" OR "long-term pain" OR "chronic primary pain" OR "chronic secondary pain" OR fibromyalgia OR "musculoskeletal pain" OR "neuropathic pain" OR "cancer pain" OR "back pain"	
**Intervention/treatment:**	
("activity scheduling" OR "pleasant activities" OR "activity engagement" OR "activity monitoring") AND (behavioral OR behavioural)	
Total records retrieved before deduplication across ClinicalTrials.gov searches: 50<br>Duplicates removed within ClinicalTrials.gov searches: 1<br>Unique ClinicalTrials.gov records exported/imported for screening: 49	

ANZCTR Search Strategy

Database/platform: ANZCTR Registry
Date searched: 5 May 2026
Limits applied: None
Search result: 276 records before deduplication across searches

Search line	Search terms	Results
**Search 1**	behavioral activation pain	19
**Search 2**	behavioural activation pain	19
**Search 3**	behavioral activation chronic pain	11
**Search 4**	behavioural activation chronic pain	11
**Search 5**	behavioral activation fibromyalgia	0
**Search 6**	behavioural activation fibromyalgia	0
**Search 7**	activity scheduling behavior pain	28
**Search 8**	activity scheduling behaviour pain	28
**Search 9**	pleasant activities behavior pain	1
**Search 10**	pleasant activities behaviour pain	1
**Search 11**	activity engagement behavior pain	25
**Search 12**	activity engagement behaviour pain	25
**Search 13**	activity monitoring behavior pain	54
**Search 14**	activity monitoring behaviour pain	54

Note: Search terms were run separately using the registry's basic search function because the ANZCTR interface did not retrieve records reliably when longer Boolean combinations were used. Quotation marks were not used in the main searches because the unquoted searches retrieved a small and manageable number of records and were considered more sensitive for registry searching. Activity-related terms were searched separately as supplementary sensitivity searches and combined with behavior/behaviour and pain terms to reduce retrieval of unrelated physical activity or monitoring studies. Retrieved records were exported where possible and imported for deduplication and screening. Relevance was assessed during the screening stage according to the eligibility criteria.

ANZCTR webpage records found across searches: 276
ANZCTR-native records exported to Excel: 262
Non-ANZCTR registry records displayed in ANZCTR search results but not exportable through the ANZCTR Excel function: 14

Total records before deduplication across ANZCTR Search Strategy searches:262
Duplicates removed within ANZCTR Search Strategy searches: 178
Unique ANZCTR records exported/imported for screening: 84

ISRCTN Search Strategy

Database/platform: ISRCTN Registry
Date searched: 5 May 2026
Limits applied: None
Search result: 11 records before deduplication across searches

Search line	Search terms	Results
**Search 1**	"behavioral activation" pain	1
**Search 2**	"behavioural activation" pain	3
**Search 3**	"behavioral activation" "chronic pain"	0
**Search 4**	"behavioural activation" "chronic pain"	1
**Search 5**	"behavioral activation" fibromyalgia	0
**Search 6**	"behavioural activation" fibromyalgia	0
**Search 7**	"activity scheduling" behavior pain	0
**Search 8**	"activity scheduling" behaviour pain	1
**Search 9**	"pleasant activities" behavior pain	0
**Search 10**	"pleasant activities" behaviour pain	1
**Search 11**	"activity engagement" behavior pain	0
**Search 12**	"activity engagement" behaviour pain	1
**Search 13**	"activity monitoring" behavior pain	0
**Search 14**	"activity monitoring" behaviour pain	3
Note: Retrieved records were exported where possible and imported for deduplication and screening. Relevance was assessed during the screening stage according to the eligibility criteria.		
Total records before deduplication across ISRCTN Search Strategy searches: 11<br>Duplicates removed within ISRCTN Search Strategy searches: 1<br>Unique ISRCTN records exported/imported for screening: 10		

WHO ICTRP Search Strategy

Database/platform: World Health Organization International Clinical Trials
Date searched: 5 May 2026
Limits applied: None
Search result: 29 records before deduplication across searches

Search line	Search terms	Results
**Search 1**	("behavioral activation" OR "behavioural activation") AND (pain OR "chronic pain" OR fibromyalgia)	18
**Search 2**	("activity scheduling" OR "pleasant activities" OR "activity engagement" OR "activity monitoring") AND (behavior OR behaviour) AND pain	11
Notes: Retrieved records were exported where possible and imported for deduplication and screening. Relevance was assessed during the screening stage according to the eligibility criteria.		
Total records before deduplication across WHO ICTRP searches: 29<br>Duplicates removed within WHO ICTRP searches: 4<br>Unique WHO ICTRP records exported/imported for screening: 25		
