# Supplementary material for: Safety and Effectiveness of Behavioural Activation for Chronic Pain: A Protocol for a Systematic Review and Meta-Analysis of Randomised Controlled Trials
Source: Nurs Rep. 2026 Aug 17;16(8):286. doi: 10.3390/nursrep16080286 (PMC13516624; doi:10.3390/nursrep16080286)
Supplement: Supplementary file 1 [file nursrep-16-00286-s001.zip › File S2-Planned Data Extraction Forms.pdf]

Data Extraction Form

Section 1. Study Identification

**Variable**	**Extracted information**
Author(s)	
Year	
Country	
Study design	
Setting	
Aim/objective	
Funding source	
Conflict of interest	
Trial registration number	
Published/unpublished/registry record	
Related reports of the same trial	

Section 2. Participant Characteristics

**Variable**	**Extracted information**
Sample size (total and per group)	
Age (mean, SD, range and age group if reported)	
Sex/gender distribution (% female)	
Type of chronic pain	
Pain duration	
Comorbidities (if reported)	

Inclusion criteria	
Exclusion criteria	
Baseline characteristics (intervention vs comparator)	

Section 3. Intervention (Behavioural Activation)

**Variable**	**Extracted information**
Intervention name	
Description of BA	
Theoretical framework (if reported)	
Activity scheduling (Yes/No/Unclear)	
Pleasant activities (Yes/No/Unclear)	
Activity monitoring (Yes/No/Unclear)	
Engagement in meaningful/pleasant activities (Yes/No/Unclear)	
Other components	
Delivery mode	
Delivery personnel	
Duration (weeks)	
Frequency	
Number of sessions	
Session length	
Intervention intensity (total hours)	
Self-directed practice (if any)	
Fidelity/adherence reported	
Adaptations to BA content, delivery, or target population, if reported	

Section 4. Comparator

**Variable**	**Extracted information**
Comparator type/category	
Description of comparator	
Comparator components, dose, or intensity (if reported)	
Co-interventions allowed or reported	

Section 5. Outcomes (Descriptive)

**Variable**	**Extracted information**
Outcome domain	
Outcome measure	
Direction of scoring	
Time points reported	
Primary outcome in the trial (Yes/No/Unclear)	
Pain intensity reported (Yes/No/Unclear)	
Additional outcomes reported, for descriptive purposes only (if applicable)	
Safety outcomes assessed (Yes/No/Unclear)	
Adverse events reported (Yes/No/Unclear)	
Serious adverse events reported (Yes/No/Unclear)	
Withdrawals due to adverse events reported (Yes/No/Unclear)	
Explicit report of no adverse events (Yes/No/Unclear)	
Safety monitoring procedures reported (Yes/No/Unclear)	
Description of adverse events, serious adverse events, withdrawals due to adverse events, and/or safety monitoring procedures	

Section 6. Outcome Data (for Meta-analysis)

Variable	Extracted information
Outcome measure	
Time point	
Follow-up category (post-intervention / short-term/medium-term / long-term)	
Type of data (change score / post-intervention score/final value)	
Intervention n analysed	
Intervention Mean	
Intervention SD	
Comparator n analysed	
Comparator Mean	
Comparator SD	
Standard error (if reported)	
Confidence interval (if reported)	
Effect estimate (if reported)	
Data required for MD/SMD available (Yes/No/Unclear)	
Data source (text/table/figure/appendix/trial registry)	
Data estimated or converted (Yes/No)	
Method used for estimation or conversion	
Notes	

Section 7. Safety Outcome Data

Variable	Extracted information
Safety outcome type (adverse events / serious adverse events/withdrawals due to adverse events)	
Time point	

Intervention events/ total n	
Comparator events/ total n	
Risk ratio calculable (Yes/No/Unclear)	
Explicit report of no events (Yes/No/Unclear)	
Safety monitoring procedures, if reported	
Notes	

Section 8. Risk of Bias (RoB 2)

**Domain**	**Judgement / supporting information**
Bias arising from the randomisation process	
Bias due to deviations from intended interventions	
Bias due to missing outcome data	
Bias in measurement of the outcome	
Bias in selection of the reported result	
Overall judgement	
Notes / justification	

Section 9. Notes and Author Contact

**Variable**	**Extracted information**
Additional notes	
Missing or unclear data	
Contact with authors required (Yes/No)	
Reason for contact	
Author contacted (Yes/No)	

Response received (Yes/No)	
Information obtained from author contact	
